# A Double-edged Sword: Balancing the Benefits and Risks of Clozapine

**DOI:** 10.1192/bjo.2025.10770

**Published:** 2025-06-20

**Authors:** Rajasee Sharma, Mohamad Arifin, Mariam Janjua, Asma Javed

**Affiliations:** Black Country Healthcare NHS Foundation Trust, Dudley, United Kingdom

## Abstract

**Aims:** Clozapine is recommended for treatment-resistant schizophrenia after two other antipsychotics have failed. We discuss a case of clozapine toxicity on therapeutic dose and its complications due to multifactorial causes.

**Methods:** 60 years Caucasian female, diagnosed with Paranoid Schizophrenia in the early 80s and on clozapine since 2000. Her mental health had been stable since and she was on 375 mg/day. There were some residual symptoms but otherwise functioning well with support. She was admitted to General Hospital with pneumonia, weight loss and altered bowel habits but nil sinister found. Her clozapine was missed for 72 hours and she developed rebound psychosis. Clozapine was re-titrated to 300 mg/day as her clozapine levels were 720 mcg/L before admission. She was discharged on the 300 mg/day however she was readmitted to General Hospital within a few months after sustaining a fracture of the femur and underwent surgery. Clozapine assays showed high levels of 1882 mcg/L with MR 2.68. She had ongoing symptoms suggestive of delirium. She was investigated for possible infections, drug interactions, and liver issues and initiated on lamotrigine for seizure prophylaxis. The clozapine dose was gradually reduced with regular monitoring of levels and side effects. She later developed stroke-like symptoms, with a normal CT scan. The clozapine dose was reduced to 100 mg/day as clozapine toxicity can mimic stroke/TIA. Repeat levels were 483 mcg/l. The patient’s mental and physical health remained stable after this.

**Results:** Clozapine is an effective antipsychotic agent with a dose range of 200 mg/day to 450 mg/day, the maximum licensed dose up to 900 mg/day. In clinical practice, the dose of clozapine is usually adjusted to provide plasma concentrations of 350–600 mcg/L. Acute infection can increase clozapine levels by reduced expression of CYP1A2 due to increased inflammatory cytokines and increase in a-1 acid glycoprotein. Acute infection and illness can lead to significantly increased clozapine levels and presumed toxicity, but symptoms of toxicity may be minimal or absent as the concentration of unbound drug is not increased. Certain drugs such as antibiotics and enzyme inducers can also affect levels.

**Conclusion:** Drug monitoring of clozapine is not a routine practice; certain clinical situations warrant multiple comorbidities, increasing age, polypharmacy, infections (pneumonia), and recent surgery, if poor (reduced) clozapine metabolism is suspected. Monitoring can help minimise the risk of toxicity. Patients should be evaluated for dose-related side effects such as constipation, seizures, cardiac arrhythmias and seizure prophylaxis should be considered if very high clozapine plasma levels.

